# Rapid Evaluation of Chemical Consistency of Artificially Induced and Natural Resina Draconis Using Ultra-Performance Liquid Chromatography Quadrupole-Time-of-Flight Mass Spectrometry-Based Chemical Profiling

**DOI:** 10.3390/molecules23081850

**Published:** 2018-07-25

**Authors:** Qianping Chen, Lili He, Changming Mo, Zhifeng Zhang, Hairong Long, Xiaoyu Gu, Ying Wei

**Affiliations:** 1 National Engineering Institute for the Research and Development of Endangered Medicinal Resources in Southwest China, Guangxi Botanical Garden of Medicinal Plants, Nanning 530023, China; chenqp79@126.com (Q.C.); longhr83@163.com (H.L.); guxiaoyu1977@hotmail.com (X.G.); weiying601@126.com (Y.W.); 2 Guangxi Crop Genetic Improvement and Biotechnology Lab, Nanning 530007, China; mochming@126.com; 3 Institute of Qinghai-Tibetan Plateau, Southwest University for Nationalities, Chengdu 610041, China; zhangzhf99@gmail.com

**Keywords:** *Dracaena cochinchinensis* (Lour.) S. C. Chen, artificially induced, Resina Draconis, dragon’s blood, UHPLC-QTOF-MS/MS

## Abstract

Resina Draconis is a highly valued traditional medicine widely used in Arabia since ancient times, and it has been commonly used as an antidiarrheic, antimicrobial, antiulcer, blood circulation promoter as well as an anti-inflammatory agent. The tree source from which this medicine orignates grows extremely slowly, producing a very low yield of Resina Draconis. To meet the increasing market demand, artificial methods for stimulating Resina Draconis formation have been developed and applied. However, the chemical differences between artificially induced Resina Draconis (*AIRD*) and natural Resina Draconis (*NRD*) have been rarely studied. The aim of this research was to explore and identify the chemical constituents of *AIRD* and *NRD* using ultra-performance liquid chromatography-quadrupole-time-of-flight mass spectrometry (UHPLC-QTOF-MS/MS) based chemical profiling. A total of 56 chromatographic peaks were detected in *AIRD*, of these, 44 peaks have had their structures tentatively characterized based on high-resolution mass spectra (HRMS) data, fragmentation ions information, reference standards data and literature review. In total, 40 peaks were found both in *AIRD* and *NRD.* The potential chemical transformation mechanisms active in Resina Draconis during formation were explored. To the best of our knowledge, this is the first evaluation of the chemical profiles of both *AIRD* and *NRD*. Furthermore, these findings are expected to provide a rational basis for the quality assessment of *AIRD* and the use of *AIRD* as a substitute for *NRD*.

## 1. Introduction

Resina Draconis (also called “dragon’s blood”), is a red resin derived from *Dracaena cochinchinensis* (Lour.) S. C. Chen and called “longxuejie” in China. It is a rare and precious traditional medicine that has been commonly used in China for the treatment of wounds, leucorrhea, fractures, diarrhea, as well as intestinal and stomach ulcers since ancient times [1]. Recent pharmacological research has shown that Resina Draconis has antithrombotic [2], antibacterial [3], anti-inflammatory [4,5], anti-diabetic [6], anti-*Helicobacter pylori* [7] bioactivity, and with the potential to be a therapeutic agent for neurodegenerative diseases [8]. Previous phytochemical studies of this resin have shown that it mainly contains phenolic compounds, including flavonoids, steroids and stilbenoids, which are considered to be the biologically active components of Resina Draconis [1]. Resina Draconis plants originate from four genera of *Dracaena*, *Daemonorops*, *Croton* and *Pterocarpus*, which are found all over the world. In China, the main source of Resina Draconis is from *Dracaena cochinchinensis* (Lour.) S. C. Chen and *Dracaena cambodiana* Pierre ex Gagnep [9]. In recent years, demand for Resina Draconis in the medicinal market has increased dramatically. However, the natural *Dracaena* tree grows extremely slowly, taking usually 30–50 years or more, and yields Resina Draconis with a very low efficiency. To meet the increasing demands for Resina Draconis, a considerable number of artificial methods for stimulating resin formation have been developed and applied [10,11]. However, a debate has continued since the emergence of artificially induced Resina Draconis (*AIRD*). The focus of this controversy is that the chemical components of *AIRD* are thought to perhaps differ from those of its natural form, which make their efficacy not equivalent. In fact, few of the chemical constituent of *AIRD* have been reported [10,12,13], however, until now, the chemical profile differences between *AIRD* and natural Resina Draconis (*NRD*) have been rarely studied. Whether the chemical constituents of *AIRD* consistent to that of *NRD* and how the secondary metabolites produced during Resina Draconis formation have are very important concerns for the efficacy and quality control of *AIRD.* Therefore, development of a generally reliable, sensitive, and confirmatory analytical method to examine the chemical constituents of both *AIRD* and *NRD* is desirable.

In recent years, tandem ultra-high-performance-liquid-chromatography with quadrupole time of flight mass spectrometry (UHPLC-QTOF-MS/MS) has been used as a rapid and effective technique to identify compounds in complex matrices. Compared with the low-resolution MS methods such as quadrupole, triple quadrupole and ion trap mass spectrometry, Q-TOF MS/MS has the ability to measure the exact mass for both precursor and fragment ions, which could be valuable for determination of structural conformation for non-target compounds in complex matrices when the reference compounds are unavailable. Thus, the present study aimed to investigate the chemical constituents in both *AIRD* and *NRD* using UHPLC-QTOF-MS/MS based chemical profiling. A salt solution containing 1% zinc sulfate and 2.0 g/L benzoic acid was used to treat the xylem of *D. cochinchinensis* to induce Resina Draconis formation by transpiration for 80 days. Then the chemical fingerprints of both *AIRD* and *NRD* were compared in both negative and positive ion modes by an improved UHPLC–QTOF-MS/MS analysis. The identities of all detected peaks, were confirmed by comparing the mass spectra and retention times with those of available reference compounds, and/or tentatively assigned by matching empirical molecular formula with those of published compounds, elucidating quasi-molecular ions and fragment ions referring to the available literature information. The potential chemical transformation mechanism of Resina Draconis during formation was further explored. The established approach was applied to rapidly identify the chemical profiles of *AIRD* and *NRD*, which offered a systematical and reliable approach for quality assessment and control of *AIRD*.

## 2. Results

### 2.1. Optimization of Chromatographic Conditions and TOFMS Method

**Figure 1:**
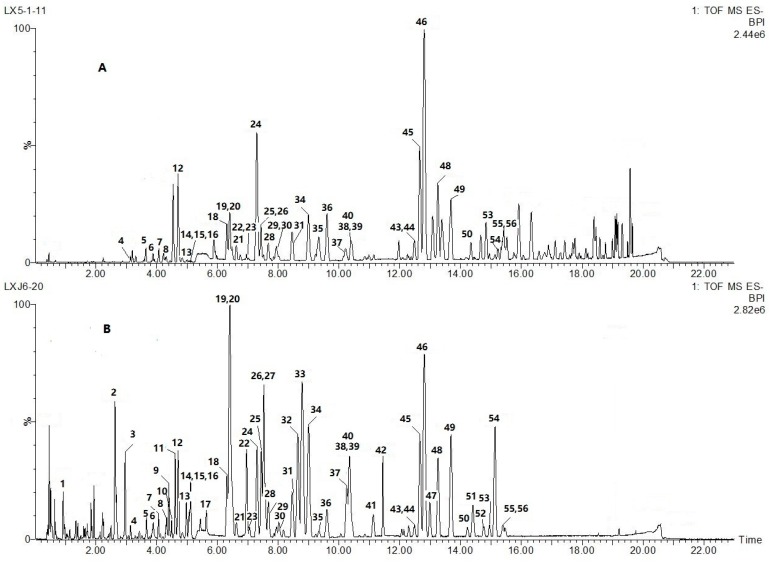
Representative basic peak ion (BPI) chromatograms of natural and artificial Resina Draconis in negative ion mode by ultra-high-performance-liquid-chromatography with quadrupole time of flight mass spectrometry (UHPLC-QTOF-MS/MS). (**A**) Natural Resina Draconis sample (*NRD*); (**B**) artificial induced Resina Draconis (*AIRD*) sample. The peak numbers are the same as displayed in Table 1.

In order to achieve a rapid and efficient analysis, the UHPLC analysis method was optimized. Different columns including Waters Acquity BEH C18 (100 mm × 2.1 mm, 1.7 m), Waters Acquity HSS T3 (100 mm × 2.1 mm, 1.8 m), Waters BEH HILIC (100 mm × 2.1 mm, 1.7 m), and different temperatures (i.e., 25, 35, 40, and 50 °C) were tested. We found that ACQUITY BEH C_18_ column (100 mm × 2.1 mm, 1.7 μm) at a temperature of 35 °C had the best baseline separation of the most of the constituents present in *AIRD* and *NRD*. Different kinds of mobile phases, including organic phase (MeOH, acetonitrile), variety of aqueous phase (water, water containing formic acid, water containing formic acid and ammonium) were tested. It was finally determined that formic acid added to the mobile phase not only improved the chromatographic peaks but also generated [M − H + HCOOH]^−^ adduction, which was helpful for the confirmation of molecular ions [M − H]^−^. Thus, a mixture system of acetonitrile-water (containing 0.1% formic acid) was finally selected to get the greatest separation, response value and better peak shapes. Different MS parameters (i.e., detection mode, capillary, cone voltage, desolvent gas flow rate, desolvent gas temperature, cone gas flow rate, and source temperature, induced dissociation energy) were investigated as well. Comparison of peak purity and a number of characteristic peaks were acquired under different ion modes. It was found that the negative-ion ESI-QTOF-MS produce more information and better signal-to-noise (S/N) ratios for active ingredients than that in positive-ion mode, so it was suitable for multi-ingredients characterization and fingerprint analysis of *AIRD* and *NRD*. Induced dissociation (CID) voltage ramping from 35 V to 50 V was selected to produce more precursor ions and fragments ions information by only one injecting sample under the MS^E^ mode. With these optimized chromatographic and MS conditions, a total 56 components could be detected in *AIRD* and *NRD* samples within 22 min. The representative chromatogram of *AIRD* (80-day inducing stage) and *NRD* obtained from the analysis in ESI negative mode is shown in Figure 1.

### 2.2. Global Characterization of the Chemical Constituents in the Artificially Induced Resina Draconis by UHPLC-Q-TOF-MS/MS

UHPLC-TOF-MS/MS was performed to screen, identify, and further characterize the constituents of *AIRD* and *NRD* in both the positive and negative ionization modes. A total of 56 compounds were clearly detected in *AIRD* using the established method (Figure 1). Of these, 44 peaks were tentatively or unambiguously characterized by matching the molecular formulas, quasi-molecular ions, and fragmentations with those of published data for the first time. Among these, seven compounds, namely 7,4′-dihydroxyflavone, protogracillin, protodioscin, 7-hydroxyflavone, loureirin D, loureirin A and loureirin B (peaks **12**, **16**, **17**, **25**, **31**, **48**, **49**) were unequivocally identified by matching the retention times and mass spectra with those of reference compounds. A total of 40 peaks were found in both *AIRD* and *NRD*. The characterization of chemical constituents based on MS fragmentation behaviors, chromatographic retention times, and literature information are summarized in Table 1.

### 2.3. Characterization of Steroid Saponins in Artificially Induced Resina Draconis

Steroid saponins are one of the major bioactive compounds presented in Dracaena species. Usually, steroid saponin compounds provide accurate structural information on the glycan sequences and the aglycone structures by UHPLC–QTOF–MS/MS method. Deprotonated ions of saponins tend to produce signals with losses of several sugar moieties in the negative mode by the successive losses of hexose, pentose, or deoxyhexose (*m*/*z* 162, 132 or 146). In this work, rapid identification of 13 steroid saponins was achieved using UHPLC–QTOF-MS/MS by matching empirical molecular formulas with those of published compounds, and/or elucidating quasi-molecular ions and fragmentations referring to the fragmentation patterns as well as available information in the literature.

**Figure 2:**
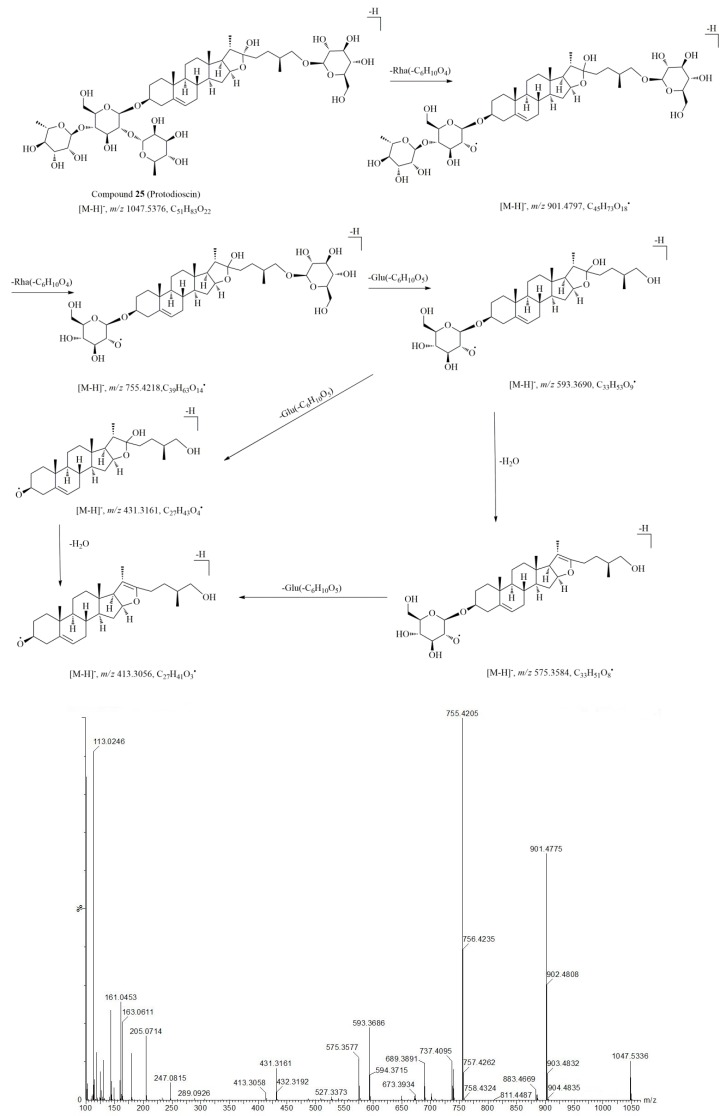
The hypothesized fragmentation pathway and MS/MS spectrum of compound **25**.

For example, Compound **25** (*t*_R_ = 7.44 min) and compound **26** (*t*_R_ = 7.53 min) were identified as steroid saponins in Resina Draconis. Compound **25** readily yielded a strong [M + HCOO]^−^ at *m*/*z* 1093.5447 in negative mode with the molecular formula C_51_H_84_O_22_. The main fragment ions at *m*/*z* 901.4797 [M − H − 146]^–^, 755.4218 [M − H − 146 − 146]^–^, 593.3641 [M − H − 146 − 146 − 162]^–^, 431.3161 [M − H − 146 − 146 − 162 − 162]^−^ corresponding to the loss of the glucosyl and rhamnosyl units (Table 1), suggesting that compound **25** contained two deoxyhexose and one hexose as a terminal unit and one hexose as an inner unit at the sugar moiety. The fragment ion *m*/*z* 413.3059 was ascribed to the aglycone ion of steroid aglycones. The postulated fragmentation pathway and MS/MS spectrum of compound **25** is shown in Figure 2. Compared with the reference and literature data, compound **25** was identified as protodioscin, which has been reported in *D. cochinchinensis* previously [14]. Compound **26** (*t*_R_ = 7.53 min) displayed a similar molecular ion at *m*/*z* 1093.5447 [M + HCOO]^–^ and a similar molecular formula, C_51_H_84_O_22_, as compound **25** in the negative ionization mode. The most abundant ion observed at *m*/*z* 901.4796 corresponds to the neutral loss of one rhamnosyl residue [M − H − 146]^–^, the other ions at *m*/*z* 755.4224 [M − H − 146 − 146]^–^, *m*/*z* 737.4127 [M − H − 146 − 146 − 18]^–^, *m*/*z* 593.3691 [M − H − 146 − 146 − 162]^–^, and *m*/*z* 575.3599 [M − H − 146 − 146 − 162]^–^ could be assigned to the subsequent loss of one and two glucosyl, rhamnosyl residues and H_2_O respectively. Furthermore, the fragmentation ions at *m*/*z* 901, 755, 737, 689, 593, 575, 431 were observed both in compound **25** and **26**. Thus compound **26** was characterized as an isomer of compound **25**, and was tentatively identified as protoneodioscin, which has also been isolated from *D. cochinchinensis* during previous study [14]. By using a similar approach, another six steroid saponin compounds (**10**, **16**, **22**, **27**, **42**, **54**) were tentatively identified through comparison with literature data combined with the structural data from other databases (Pubmed, Mass Bank, Chemspider, etc.)

### 2.4. Characterization of Flavonoid Compounds in Artificially Induced Resina Draconis

**Figure 3:**
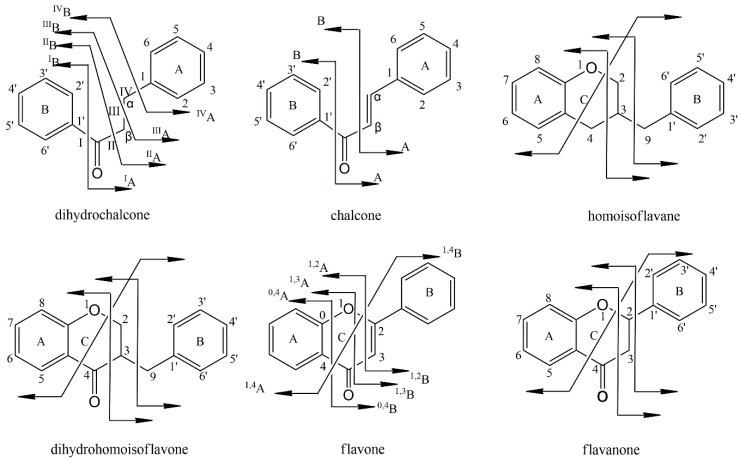
Fragmentation pathway of flavonoid compounds in Resina Draconis.

The flavonoid compounds observed in *AIRD* were mainly categorized into several subtypes, such as flavones, flavanones, chalcones, dihydrochalcones, homoisoflavanes. From the results of the QTOF-MS/MS mass spectra, we can see that most of the flavonoids compounds had strong responses under negative ion scan modes and easily gave quasi-molecular ion peaks of [M − H]^–^, [M + HCOO]^–^ and [2M − H]^–^. The fragmentation rules of flavonoids facilitate their structure identification, such as the neutral losses of CO (**28**), due to the contraction of the ring with carbonyl group, the loss of H_2_O (**18**) implies the presence of a hydroxyl group, and the losses of CH_3_ (**15**), OCH_3_ (**31**) explain the presence of a methoxyl group. The proposed fragmentation pathways of flavonoid compounds presented in *AIRD* and *NRD* are summarized in Figure 3. Usually, flavanes and flavanones easily utilize the Retro–Diels–Alder (RDA) fragmentation pathway. Fragment ions derived from RDA fragments are more abundant than the loss of other radical ions, for example, the loss of CH_3_, CO, OH or H_2_O. On the other hand, chalcones, dihydrochalcones, flavanes as well as homoisoflavanes have very weak RDA fragmentation. Their main fragmentation pathway is the breakage of C_3_–C_9_ bond, which lead to the loss of the B-ring. The structures of homoisoflavanoids are special because of an additional methylene linking between B− and C−rings. The cleavages of C_3_–C_9_ and/or C_9_–C_1′_ leading to loss of B−ring, were regarded as diagnostic fragments for this kind of flavonoids. For chalcones, cleavage beside the carbonyl group usually occurs because there is a C=C double bond at the α-β position, while dihydrochalcones, with a saturated C−C backbone, always encounter the breakage of the C−C bone at the α−β position.

**Figure 4:**
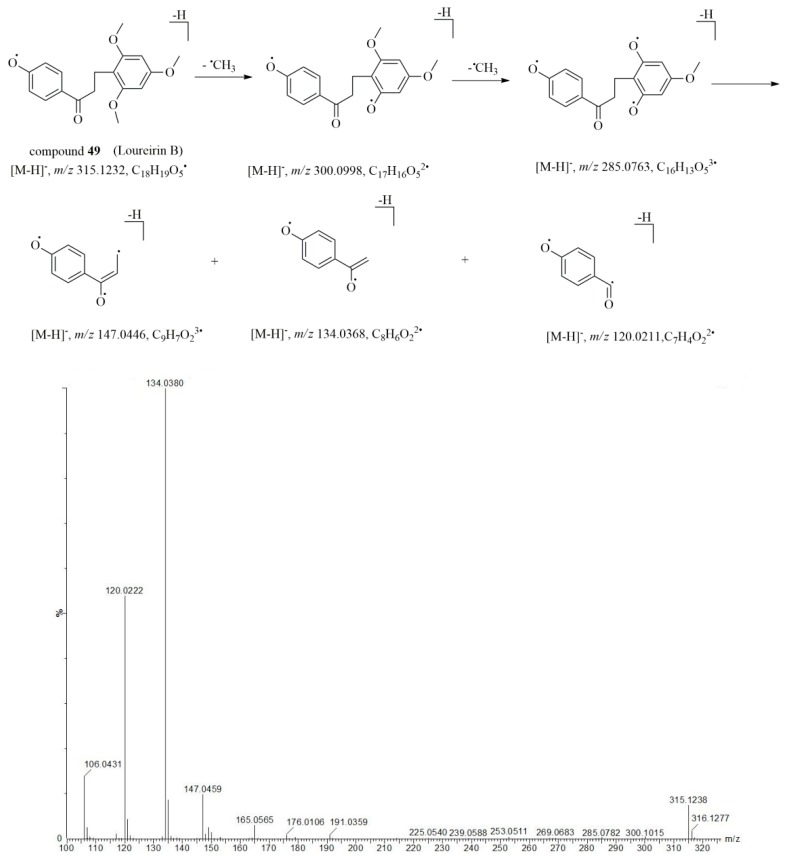
The hypothesized fragmentation pathway and MS/MS spectrum of compound **49**.

The prominent fragmentation pathways proposed here were expected to facilitate the characterization of flavonoid compounds in *AIRD* and *NRD*. Thus, by using the method described above, a total of 36 flavonoid compounds in *AIRD* were identified. For example, as a typical dihydrochalcone, the deprotonated molecularion of compound **49** (*t*_R_ = 13.69 min) was observed at *m*/*z* 315.1229 [M − H]^–^ under negative ionization mode. The product ions of *m*/*z* 300.0998 [M − H − CH_3_]^–^ and 285.0801 [M − H − 2CH_3_]^−^ corresponded to the radical cleavage of the methyl group, and the fragmentations at *m*/*z* 147.0450, 134.0370 and 120.0212 contributed to the successive neutral loss of the B ring at the II, III and IV bond positions, respectively. The characteristic fragments, primary cleavages, and MS/MS spectrum of compound **49** are elucidated in Figure 4. Compared with reference and literature data [13,26], compound **49** was identified to be loureirin B. It is interesting to note that there were common fragment ions observed at most of the dihydrochalcone compounds at *m*/*z* 147.05, 134.04 and 120.02, which could due to the successive neutral loss of the B ring at the positions of II, III and IV bond. For example, compounds **17** (*t*_R_ = 5.64 min), **28** (*t*_R_ = 7.68 min), **37** (*t*_R_ = 10.26 min) and **48** (*t*_R_ = 13.69 min) displayed molecular ions at *m*/*z* 287.0914 [M − H]^–^, 257.0809 [M − H]^–^, 301.1077 [M − H]^–^ and 285.1123 [M − H]^–^, respectively in the negative mode. With the assistance of formula predictor software, the elemental composition of the sequasi-molecular ions was calculated to be C_16_H_16_O_5_, C_15_H_14_O_4_, C_17_H_18_O_5_ and C_17_H_18_O_4_. From the MS/MS spectrum (Table 1), characteristic fragments were observed at *m*/*z* 147.05, 34.04 and 120.02 in compounds **17, 28, 37**, and **48**, respectively, suggesting these compounds are derivatives of typical dihydrochalcones. Thus, by comparing these fragmentation behaviors with those of the reference compounds, MS/MS fragmentation data and literatures, compounds **17, 28, 37** and **48** were tentatively identified as loureirin D [13], 2,4,4′-trihydroxydihydrochalcone [18], 4,4′-dihydroxy-2,6-dimethoxydihydrochalcone [16] and loureirin A [13, 26], respectively.

### 2.5. Chemical Transformation of Artificially Induced Resina Draconis and Natural Resina Draconis

Qualitative and semi-quantitative comparisons of chemical constituents between *AIRD* and *NRD* samples were carried out using our newly established UHPLC-QTOF-MS/MS method. The relative peak height of steroid saponins **22**, **25**, **26**, **27**, **42** and **54** detected in *AIRD* were significantly higher than those in *NRD*. It has been reported that the chemical constituents of fresh stems from *D. cochinchinensis* trees were quite different from the constituents of their xylem resins [10]. More than 60% of compounds isolated from fresh stems were steroid saponins, and the flavonoids compounds were undetectable in fresh stem xylem. However, flavonoid content increased dramatically after wounding and/or fungal infection of these plants [10]. Conversely, steroid saponins decreased considerably along with infection time. It was hypothesized that the wound-activated defense response of trees result in Resina Draconis formation; this phenomenon is thought to be a defense response of plants against attack by foreign bodies. Wounding triggers the biosynthesis of flavonoids components through the phenylpropanoid pathway during the defensive reaction process [10]. Flavonoid content was positively correlated with infection time while the steroid saponin content was negatively correlated. All of the above results are in agreement with reports from the literature. Furthermore, it was found that, the number and intensity of higher polarity components in *AIRD*, eluted before 6 min at the BPI chromatograms (Figure 1), were more abundant than that those of *NRD*. While the lower polarity components, eluted after 16 min, were increased significantly in *NRD* but decreased markedly or even not detected in *AIRD*. These lower polarity compounds, identified as flavonoid dimers according to their molecular formulas, and related literatures, had been reported in *NRD* in a previously published report [28]. Interestingly, the composition of the phenolic substances in *AIRD* was dramatically different from that of *NRD*. The relative peak heights of flavonoids compounds (**19**, **20**, **32**, **33**, **34**, **38**, **39**, **40**, **41**) detected in *AIRD* were higher than those of *NRD*. This phenomenon could be attributed to the formation and conversion of the simple flavonoids with endogenous enzymes and/or the metabolism by exogenous microorganisms during the production of the resin [28,29]. It has been reported that dihydrochalcone compounds are one of the main components of Resina Draconis, and could be polymerized further with the other flavones components to form dimer, trimer, and tetramer compounds [28,29], such content may reach 20% of the total mass accompanying the formation of Resina Draconis. In this study, the accumulation of Resina Draconis only took 80 days and during that time a considerable amount of flavonoids were synthesized, however, no flavonoid oligomers were detected by MS. It is likely that the induction time of Resina Draconis was too short to oxidize the dihydrochalcone and flavones further to form flavonoid oligomers.

## 3. Materials and Methods

### 3.1. Materials and Reagents

Wild *D. cochinchinensis* samples were collected from authenticated locations in Ningming county, Guangxi province, China, the indigenous wild region of this plant. These voucher specimens, identified by Professor Baoyou Huang, have been deposited in the Herbarium Centre, Guangxi Botanical Garden of Medicinal Plants, Nanning, China. Five year-old *D. cochinchinensis* saplings were approximately 1.5 m in height, with a diameter of 4.0–6.0 cm at 10 cm above ground. The reference compounds 7,4′-dihydroxyflavone, protogracillin, protodioscin, 7-hydroxyflavone, loureirin D, loureirin A and loureirin B were purchased from the National Institutes for Food and Drug Control (Beijing, China). All of the chemicals were obtained with a purity of 98% or above, as confirmed by normalization of the peak area detected by UHPLC-QTOF-MS (Waters Corporation, Milford, MA, USA). HPLC-grade acetonitrile, methanol, and formic acid (purity: 98%) were purchased from E. Merck (Darmstadt, Germany). Ultra-pure water was obtained from a Milli-Q water purification system (Millipore, Bedford, MA, USA). Other solvents and chemicals were of analytical grade and purchased from the Guangdong Guanghua Sci-Tech Co., Ltd. (Shantou, China).

### 3.2. Induction of D. cochinchinensis Saplings to Produce Resina Draconis

**Figure 5:**
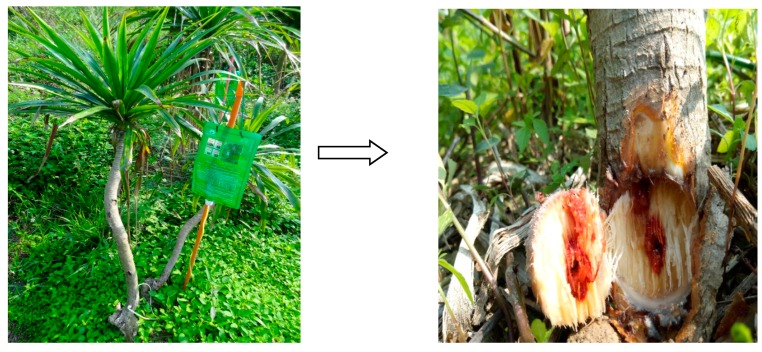
Resina Draconis produced in the stem xylem of *D. cochinchinensis* trees after chemical solution induction for 80 days.

A small hole (0.4 cm in diameter, 10 cm above ground) was drilled into the xylem stem of a wild *D. cochinchinensis* sapling, and an injector containing a salt solution (1% zinc sulfateand 2.0 g/L benzoic acid) was inserted into the hole. The salt solution was left to flow freely into the xylem via transpiration. The observed flow rate was approximately 20 mL/h. A transfusion of double distilled water was used as a control. After treating the tree for 80 days, a tissue core about 1.5 cm in diameter of the inoculated sapling xylem was collected by an electric perforator (Figure 5).

### 3.3. Preparation of Sample and Standard Solutions

The preparation of the sample solutions for the chemical profile analyses was performed as follows: six batches of red resin collected from induced *D. cochinchinensis* and nature *D. cochinchinensis* were combined respectively to get enough amounts of *AIRD* and *NRD* sample. Then, the samples were dried in an oven at 60 °C for 2 h. The dried *AIRD* and *NRD* samples were cut into small sections and ground to a fine powder in a mill. Samples were then accurately weighed powder (0.2 g) was suspended in 20 mL of 80% methanol (*v*/*v*) in a 25 mL volumetric flask and sonicated (Kun Shan Ultrasonic Instruments Co., Ltd., Kun Shan, China) for 30 min at room temperature. Then, the final volume was made up to 25 mL with 80% methanol (*v*/*v*). The supernatant of the extracts was filtered through a 0.22 μm PTFE syringe filter before UHPLC-QTOF-MS/MS analysis. Three replicates were prepared for each sample.

Stock solutions: A certain amount of 7,4′-dihydroxyflavone, protogracillin, protodioscin, 7-hydroxyflavone, loureirin D, loureirin A and loureirin B dissolved in 80% methanol-water (*v*/*v*), to create reference compound stock solutions (about 1.0 mg/mL), which was stored under 4 °C. Both solutions were filtered by a 0.22 μm PTFE syringe filter before being subjected to UHPLC-QTOF-MS analysis.

### 3.4. Qualitative Analysis by UHPLC-QTOF-MS/MS

Detection was performed using a Waters ACQUITY UHPLC ^TM^ system (Waters Corporation, Milford, MA, USA). Chromatography separation was performed with an ACQUITY BEH C_18_ column (100 mm × 2.1 mm, 1.7 μm; Waters, Milford, MA, USA), coupled with a C_18_ pre-column (2.1 mm × 5 mm, 1.7 μm, Van-Guard^TM^ BEH, Waters, Milford, MA, USA.) and a column temperature of 35 °C. The mobile phase was a mixture of 0.1% formic acid-water (A) and acetonitrile (B), with an optimized linear gradient elution as follows: 0–0.5 min, 10% B; 0.5–1.0 min, 10–18% B; 1.0–4.5 min, 18–27% B; 4.5–10 min, 27–32% B; 10–11.0 min, 38–45% B; 11.0–16.0 min, 38–45% B; 16.0–18.0 min, 45–55% B; 18.0–20.0 min, 55–85% B; 20.0–20.1 min, 85–10% B; 20.1–23.0 min, 10–10% B. The injection volume was 2 μL and the flow rate was set at 0.50 mL/min.

Mass spectrometry was performed on a definition accurate mass quadrupole time-of-flight (Q-TOF) Xevo G2-S mass spectrometer (Waters MS Technologies, Manchester, UK) equipped with electrospray ionization (ESI) source. The ESI source of the MS was operated in both positive and negative modes. All MS data were produced using LockSpray™ to ensure mass accuracy and reproducibility. The Leucine-enkephalin ions, [M − H]^−^ and [M + H]^+^ at mass to charge ratio *m*/*z* 554.2615 and *m*/*z* 556.2771 were used as the lock mass in negative and positive electrospray ionization modes, respectively. Mass spectrawere acquired in the negative ion mode by scanning from 100 to 1500 Da with a 0.20 s scan time and a 0.01 s inter-scan delay over a 22 min analysis time. MS analysis was conducted under the following operating parameters: The desolvation gas flow rate was 800 L/h at a temperature of 400 °C, and the cone gas was 20 L/h and the source temperature was 100 °C; the capillary voltage and cone voltage were set at 2400 V and 40 V, respectively. The energies for collision induced dissociation (CID) were 6 V for the precursor ion and 35–55 V for fragmentation information.

UHPLC data analysis and accurate mass as well as elemental composition were analyzed with the MassLynx 4.1 software (Waters Co., Mil-ford, CT, USA). Structural elucidation was performed by the MassFragment tool provided by MassLynx 4.1.

## 4. Conclusions

In the present study, a rapid and sensitive UHPLC-QTOF-MS/MS method was carried out to compare and identify the chemical constituents of the *AIRD* and *NRD* for the first time. A total of 44 compounds were unequivocally or tentatively identified and 40 peaks were found both in *AIRD* and *NRD*. The chemical profiles of *AIRD* and *NRD* were further explored to find the differences in secondary metabolites. Dihydrochalcone was the main bioactive component in both *AIRD* and *NRD*, which was shown to form dimer, trimer, and tetramer compounds with the other flavones through oxidation along with the formation of Resina Draconis. Oligomer of dihydrochalcone could be found in *NRD* but not in *AIRD*, which may due to an insufficient induction time of Resina Draconis (only 80 days). The association between the chemical transformation of Resina Draconis and induction time as well as the effect on the bioactivities of *AIRD* and *NRD* needs to be further explored. In short, this study clearly demonstrated the chemical constituents of *AIRD* and *NRD*, thus providing a rational basis for *AIRD* for use as a substitute for *NRD* to meet the market demand of Resina Draconis. Additionally, these results should be very useful for the quality assessment of this valuable medicine.

## Figures and Tables

**Table 1:** Components identified from artificial induced Resina Draconis using UHPLC-QTOF-MS/MS.

No.	*t*_R_/min	[M − H]^–^	Error/ppm	MS/MS Fragments Ions	Empirical Formula	Identification	Reference Literatures
**1**	0.9	351.1284	–0.2	296.9812, 289.1299, 207.0851, 161.0435, 125.0228, 113.0248	C_14_H_24_O_10_	unknown	
**2**	2.63	413.1445	–0.7	269.1028, 217.0434, 161.0453, 143.0343, 125.0249, 113.0246	C_19_H_26_O_10_	unknown	
**3**	2.96	413.1437	–2.7	269.1028, 217.0434, 161.0453, 143.0343, 125.0249,113.0246	C_19_H_26_O_10_	unknown	
**4** *	3.14	487.1818 (M + HCOO^–^)	0.4	441.1693, 307.1003, 247.0826, 214.0602, 163.0604, 145.0508, 133.0649, 125.0239	C_21_H_30_O_10_	unknown	
**5** *	3.66	1129.4753	0.1	983.4068, 967.4224, 866.0862, 784.9418, 762.1688, 696.7462, 645.0305, 496.3572	C_46_H_82_O_31_	saponins	
**6** *	3.88	967.4213	–2.2	821.3612, 788.3539, 741.3986, 591.3541, 447.3194, 357.0483, 150.9695	C_40_H_72_O_26_	saponins	
**7** *	4.05	817.422 (M + HCOO^–^)	–2.2	663.2785, 261.0729, 168.0311, 173.0305, 165.0199	C_39_H_64_O_15_	Saponins (spirostanol monodesmosides)	
**8** *	4.33	689.3203	4.4	671.3118, 527.2696, 411.1845, 369.1389, 327.1284, 233.0507, 161.1837, 150.4524	C_36_H_50_O_13_	unknown	
**9**	4.4	933.4703 (M + HCOO^–^)	0.1	741.4053, 723.3961, 609.3652, 579.3525, 465.3005, 447.3121, 429.3012, 403.2820, 161.0430, 159.0315, 143.0380, 113.0242	C_45_H_74_O_20_	saponins	
**10**	4.49	1107.5237 (M + HCOO^–^)	1.3	915.4579, 769.3976, 607.3532, 589.3440, 541.3199, 247.0806, 179.0542, 161.0447, 143.0329, 131.0348, 113.0233	C_51_H_82_O_23_	Dracaenoside Q	[14]
**11**	4.6	787.4115 (M + HCOO^–^)	–0.8	609.3638, 447.3108, 429.3003, 403.2857, 179.0560, 161.0455, 159.0297, 143.0339, 131.0338, 119.0346, 113.0239	C_38_H_62_O_14_	Saponins	
**12**	4.7	253.0499	–0.8	235.0349, 224.0459, 208.0515, 196.0493, 180.0578, 135.0086, 117.0346	C_15_H_10_O_4_	7,4′–dihydroxyflavone **	[15]
**13** *	4.84	281.0812	–0.7	265.0502, 237.0547, 221.0622, 209.0598, 194.0729, 181.0645, 167.0500, 149.0234	C_17_H_14_O_4_	5,4′-dimethoxy-7-hydroxyflavylium ^#^	[16]
**14** *	4.97	299.0912	–2.3	193.0507, 178.0277, 149.0605, 139.0403, 117.0344	C_17_H_16_O_5_	5,7,4′-trihydroxy-6-methyl-dihydrohomoisoflavone	[13]
**15** *	5.07	283.0603	–1.1	268.0374, 240.0429, 211.0405, 196.0546, 183.0444, 135.0089	C_16_H_12_O_5_	thevetiaflavone	[16]
**16** *	5.11	1109.5398 (M + HCOO^–^)	–0.7	917.4685, 901.4780, 755.4103, 593.3311, 247.0828, 199.0430, 179.0604, 163.0623, 119.0364, 113.0261	C_51_H_84_O_23_	protogracillin **	[14]
**17**	5.64	287.0914	–1.7	271.0491, 177.0198, 151.0403, 147.0445, 134.0370, 124.0161, 120.0211, 108.0219	C_16_H_16_O_5_	loureirin D **	[13]
**18** *	6.31	269.0811	–1.1	253.0495, 225.0549, 211.0393, 161.0240, 136.0159, 117.0338	C_16_H_14_O_4_	4,4′- dihydroxy-2′-methoxyl-chalcone	[17]
**19** *	6.41	269.0812	–0.7	253.0486, 229.4882, 209.0552, 163.0389, 148.0151, 135.0073, 119.0496, 109.0280	C_16_H_14_O_4_	2,4′-dihydroxy-2′-methoxychalcone	[15]
**20** *	6.48	269.0810	–1.5	253.0464, 237.0530, 201.4554, 163.0385, 148.0148, 135.0431, 119.0487, 109.0276	C_16_H_14_O_4_	7,4′-dihydroxyhomoisoflavanone	[16]
**21** *	6.62	269.0811	–1.1	253.0477, 225.0581, 211.0390, 163.0400, 148.0165, 135.0450, 119.0485, 109.0270	C_16_H_14_O_4_	2′,4′-dihydroxy-2-methoxylchalcone	[16]
**22** *	6.96	947.484 (M + HCOO^–^)	0.3	755.4211, 739.4275, 593.3695, 575.3596, 431.3154, 413.3112, 247.0826, 179.0577, 163.0603, 143.0346, 131.0364, 119.0330	C_45_H_74_O_18_	trigofoenoside A ^#^	
**23** *	7.05	271.0967	–1.1	253.0498, 187.0408, 177.0198, 165.0199, 151.0041, 145.0299, 119.0504, 107.0138	C_16_H_16_O_4_	7,3,4′-trihydroxy-8-methyl-flavane	[13]
**24** *	7.30	271.0965	–1.8	253.0517, 243.0705, 225.0564, 215.0696, 197.0617, 185.0628, 161.0618, 151.0043, 135.0458, 120.0221	C_16_H_16_O_4_	7,4′-dihydroxy-3′-methoxyl-flavane	[13]
**25** *	7.44	1093.5447 (M + HCOO^–^)	1.1	901.4803, 755.4205, 737.4112, 689.3853, 671.3862, 606.6843, 593.3641, 575.3621, 460.5794, 431.3161, 413.3059, 349.1158, 163.0628	C_51_H_84_O_22_	protodioscin **	[14]
**26** *	7.53	1093.5413 (M + HCOO^–^)	1.2	901.4796, 755.4224, 737.4127, 689.3885, 593.3691, 575.3599, 431.3156, 179.0574, 163.0611, 143.0357, 119.0354, 113.0241	C_51_H_84_O_22_	protoneodioscin	[14]
**27**	7.58	1079.5287 (M + HCOO^–^)	0.2	901.4780, 887.4639, 739.4208, 689.3886, 593.3665, 577.3621, 431.3163, 340.1174, 247.0835, 205.0723, 179.0560, 163.0625, 131.0351, 119.0346, 113.0249	C_50_H_82_O_22_	desgalactotigonin ^#^	
**28** *	7.68	257.0809	–1.9	151.0398, 147.0452, 135.0078, 134.0374, 120.0215, 109.0283	C_15_H_14_O_4_	2,4,4′-trihydroxydihydrochalcone	[18]
**29** *	7.94	301.1075	–0.3	285.0396, 268.0385, 257.0410, 241.0503, 213.0545, 199.0385, 185.0575, 164.0116, 151.0039, 136.0164	C_17_H_18_O_5_	2,4,4′-trihydroxy-3′-methoxyl-3-methyl-dihydrochalcone	[13]
**30** *	8.02	301.0707	–1.7	285.0409, 268.0372, 258.0515, 242.0588, 174.0330, 164.0129, 151.0038, 136.0139	C_16_H_14_O_6_	3,2′,3′,4′-tetrahydroxy-4-methoxyl-chalcone	[13]
**31** *	8.46	237.0546	–2.5	208.0531, 193.0663, 180.0579, 165.0709, 153.0710, 143.0498, 135.0087, 132.0213	C_15_H_10_O_3_	7-hydroxyflavone **	[19]
**32**	8.64	299.0915	–1.3	281.0819, 266.0580, 178.0275, 163.0040, 136.016, 108.0216	C_17_H_16_O_5_	7,3′-dihydroxy-4′-methoxyl-dihydrohomoisoflavone	[13]
**33**	8.78	285.0758	–1.8	270.1771, 268.9290, 252.2793, 246.3228, 179.0353, 151.0036, 135.0444, 122.0376	C_16_H_14_O_5_	3,2′,4′-trihydroxy-4-methoxyl-chalcone	[20]
**34** *	9.00	285.0758	–1.8	267.0646, 243.0655, 215.0707, 199.0771, 187.0404, 165.0198, 119.0507, 121.0301	C_16_H_14_O_5_	7,4′-dihydroxy-5-methoxyflavanone	[16]
**35** *	9.34	283.0601	–1.8	265.0530, 250.0248, 239.0720, 224.0503, 215.0722, 163.0407, 135.0460, 121.0299	C_16_H_12_O_5_	3′,7-dihydroxy-4’-methoxylflavone	[21]
36 *	9.60	315.0862	–2.2	299.0552,191.0345,178.0281,165.191,150.0320,134.0373,121.0303,108.0222	C_17_H_16_O_6_	3,7,4′-trihydroxy-5-methoxy homoisoflavanone	[16]
**37** *	10.26	301.1077	0.3	271.1297, 207.0655, 177.0197, 164.0488, 153.0560, 147.0453, 134.0457,120.0220	C_17_H_18_O_5_	4,4′-dihydroxy -2,6-dimethoxydihydrochalcone	[16]
**38** *	10.32	285.0759	–1.4	269.0446, 241.0507, 200.0438, 197.0602, 177.0190, 165.0181, 119.0482	C_16_H_14_O_5_	7,4′-dihydroxy-3′-methoxyflavanone	[16]
**39** *	10.34	315.0865	–1.3	297.0767, 282.0535, 189.0201, 178.9994, 152.0123, 124.0174	C_17_H_16_O_6_	5,7-dihydroxy-2′,4′-dimethoxyl-isoflavanone	[13]
**40** *	10.42	239.0709	0.2	221.0603, 211.0737, 197.0606, 169.0661, 148.0173, 135.0084, 120.0208, 109.0279	C_15_H_12_O_3_	7-hydroxyflavanone	[19,22]
**41**	11.13	281.0808	–2.5	251.0500, 237.0548, 221.0609, 209.0602, 193.0660, 160.0160, 153.0180, 135.0083	C_17_H_14_O_4_	5,7-dimethoxyflavone	[23]
**42**	11.44	1135.5547 (M + HCOO^–^)	–0.2	1047.5375, 1029.5267, 943.4902, 901.4797, 883.4693, 755.4202, 737.4052, 689.3869, 673.3993, 593.3668, 575.3519, 497.2161, 431.3281, 413.3172, 247.0783, 205.0778, 179.0491, 163.0622	C_53_H_86_O_23_	spongioside B ^#^	[24]
**43** *	12.48	253.0405	1.6	237.0549, 209.0597, 193.0652, 161.0239, 136.0158, 120.0211	C_15_H_10_O_4_	5,7-dihydroxyflavone	[16]
**44** *	12.48	283.0965	–1.8	268.0368, 241.0054, 197.0655, 161.0236, 146.0345, 134.0363, 120.0215, 106.0423	C_17_H_16_O_4_	5,7-dimethoxyflavanone	[16]
**45** *	12.66	253.0860	–2.0	237.0554, 209.0608, 193.0658, 161.0243, 136.0166, 120.0214, 108.021	C_16_H_14_O_3_	7-methoxydihydroflavone	[25]
**46** *	12.80	255.0656	–0.4	213.0561, 185.0613, 171.0456, 164.0122, 151.0041, 145.0662, 136.0166, 107.0139	C_15_H_12_O_4_	7,4′-dihydroxyflavanone	[13]
**47**	12.99	329.1019	–1.8	311.0912, 296.0675, 208.0363, 193.0130, 190.0261, 166.0257, 138.0305, 121.0276	C_18_H_18_O_6_	cambodianol	[13]
**48** *	13.26	285.1123	–1.4	270.0906, 257.0449, 242.0938, 229.0582, 147.0449, 134.0369, 120.0212, 106.0419	C_17_H_18_O_4_	loureirin A **	[13,26]
**49** *	13.69	315.1229	–1.0	299.0902, 283.0934, 253.0513, 191.0337, 165.0559, 147.0450, 134.0370, 120.0212	C_18_H_20_O_5_	loureirin B **	[13,26]
**50** *	14.22	315.1232	0.0	300.0907, 283.0933, 206.0605, 191.0368, 165.0552, 147.0471, 134.0449, 120.0205	C_18_H_20_O_5_	unknown	
**51**	14.41	297.0763	0.0	281.0450, 265.0530, 253.0509, 237.0547, 225.0542, 209.0599, 195.0456, 176.0114, 151.0037, 130.0424, 125.0247, 107.0136	C_17_H_14_O_5_	7,4′-dihydroxy-5-mehtoxy-8-metllylflavone	[23]
**52**	14.76	299.0915	–1.0	284.0689, 255.0996, 193.0474, 178.0253, 150.0305, 122.0355	C_17_H_16_O_5_	7-methoxy-5,4′-dihydroxy-8-methylflavanone	[16]
**53** *	14.95	585.2125 (M + HCOO^–^)	0.7	429.1734, 405.1335, 387.1245, 285.1133, 281.0823, 253.0856, 163.0389, 151.0422, 147.0450, 133.0667	C_33_H_32_O_7_	flavonoid dimers	
**54** *	15.13	929.4763	0.0	737.4115, 591.3526, 525.3197, 429.3015, 349.1093, 247.0842, 205.0735, 163.0618	C_45_H_72_O_17_	cambodianoside C	[27]
**55** *	15.38	239.0706	–0.8	221.0590, 211.0755, 197.0598, 169.0648, 148.0149, 135.0071, 120.0195, 109.0279	C_15_H_12_O_3_	6-hydroxyflavanone	[16]
**56** *	15.47	269.0807	–2.6	251.0717, 241.0869, 227.0703, 165.0177, 155.0852, 150.0308, 137.0219, 121.0277	C_16_H_14_O_4_	cardamomin	[16]

Note: Compounds marked with * were found in both *AIRD* and *NRD*, compounds marked with # were identified in Resina Draconis for the first time and compounds marked with ** were unambiguously identified with reference.
